# Penetration Characteristics of Air, Carbon Dioxide and Helium Transverse Sonic Jets in Mach 5 Cross Flow

**DOI:** 10.3390/s141223462

**Published:** 2014-12-08

**Authors:** Erinc Erdem, Konstantinos Kontis, Selvaraj Saravanan

**Affiliations:** 1 Aerospace Sciences Division, School of Engineering, University of Glasgow, Glasgow G12 8QQ, UK; E-Mail: erincerdem@gmail.com; 2 Department of Aerospace Eng'rg, Indian Institute of Science, Bangalore 560012, India; E-Mail: saravan@aero.iisc.ernet.in

**Keywords:** Flows and jets through nozzles, velocity measurements, visualization and imaging

## Abstract

An experimental investigation of sonic air, *CO*_2_ and Helium transverse jets in Mach 5 cross flow was carried out over a flat plate. The jet to freestream momentum flux ratio, *J*, was kept the same for all gases. The unsteady flow topology was examined using high speed schlieren visualisation and PIV. Schlieren visualisation provided information regarding oscillating jet shear layer structures and bow shock, Mach disc and barrel shocks. Two-component PIV measurements at the centreline, provided information regarding jet penetration trajectories. Barrel shocks and Mach disc forming the jet boundary were visualised/quantified also jet penetration boundaries were determined. Even though *J* is kept the same for all gases, the penetration patterns were found to be remarkably different both at the nearfield and the farfield. Air and *CO*_2_ jet resulted similar nearfield and farfield penetration pattern whereas Helium jet spread minimal in the nearfield.

## 1. Introduction

Transverse jet injection into supersonic/hypersonic crossflows has been encountered in many engineering applications ranging from scramjet combustors and solid rocket motor or liquid engine thrust vector control systems to high speed flying vehicle reaction control jets. The interaction of the transverse jet with the high speed cross flow creates complex three dimensional flow patterns commonly including separated regions, shock waves, shear layers and wakes, *etc*. The resultant flowfield has received significant interest since the 1960's. Earlier studies were focused on wind tunnel experiments utilising conventional measurement techniques such as schlieren/shadowgraph photography, wall pressure and concentration measurements to gain a better understanding of the jet interaction and penetration phenomena [1–6]. These studies aimed to assess the effect of injection pressure ratio, the location of injection, the state of the incoming boundary layer and type of injectant gas on jets in supersonic/hypersonic cross flow phenomena.

**Figure 1.**
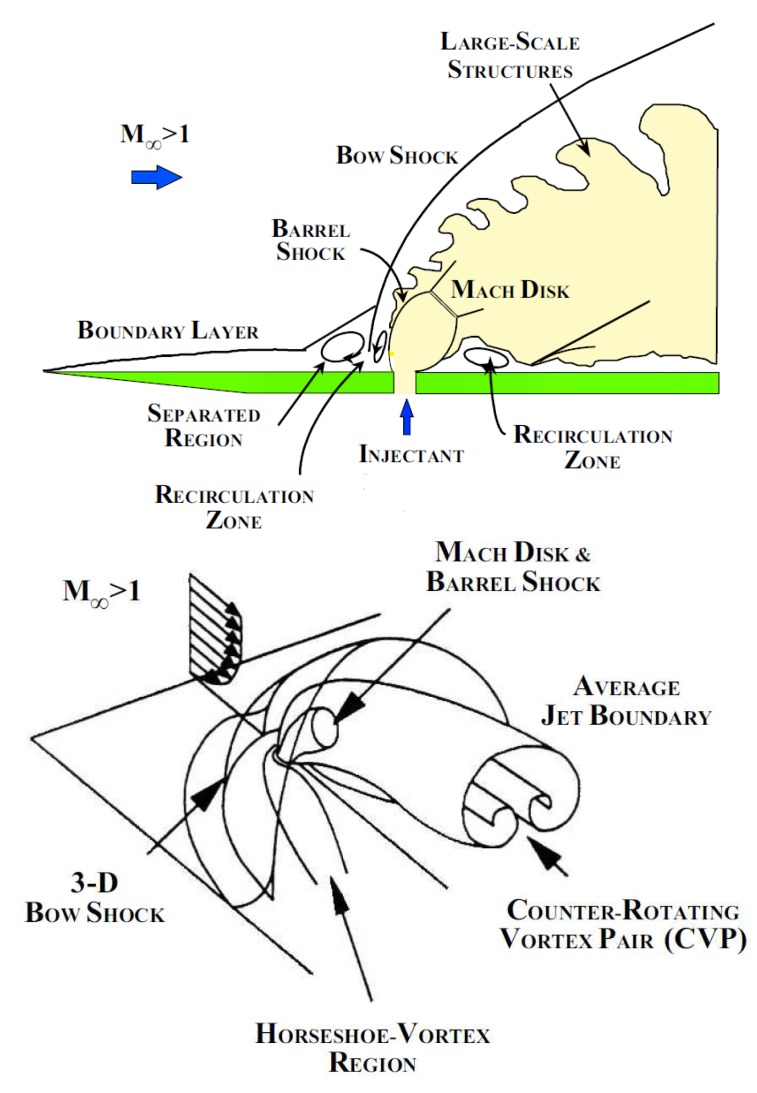
**Top**: mixing features of an underexpanded transverse injection into a supersonic cross flow; **bottom**: three dimensional perspective of the averaged features of the flowfield by [10].

On the other hand several studies investigated mixing performance and penetration characteristics of transverse jets in high speed cross flows at relatively low supersonic Mach numbers in “cold” wind tunnels [7–9]. These studies provided valuable experimental data shedding light on the jet penetration and associated trajectories measured by Mie/Rayleigh scattering. Specifically, Ben-Yakar [10] investigated the convection and mixing characteristics of hydrogen and ethylene transverse jets at Mach 3.4 in an expansion tube with realistic upstream conditions for scramjet applications. Ultra high speed schlieren photography and Planar Laser Induced Fluorescence (PLIF) of *OH* radicals were utilised to obtain detailed information on the molecular mixing. The mechanism behind the mixing was found to be the promotion of small scale structures rather than global manipulations of the main stream since mixing leading to chemical reactions occurs at the molecular level. The near field mixing of transverse jets is dominated by so-called “entrainment-stretching-mixing process” [10], driven by large scale jet shear layer vortices as they are shown in Figure 1 (top figure). Due to the obstruction introduced by transverse jet, a bow shock forms and stands off from the injector orifice. In the region between the bow shock and the transverse jet, the injectant fluid moves with a higher velocity tangentially to the interface than the free stream fluid. As a result, large vortices are periodically formed engulfing large quantities of free stream fluid and drawing it into the jet shear layer (macro-mixing). These vortices are convected downstream at high speeds, where the injectant and air are then mixed by slow molecular diffusion. They stem from so-called Kelvin-Helmholtz instabilities [11]. In general, large scale structures are beneficial for the enhancement of bulk mixing, but they hinder fine scale or molecular mixing. However, they also stretch the interface between unmixed fluids. Stretching increases the interfacial area and simultaneously steepens the local concentration gradients along the entire surface while enhancing the diffusive micro mixing [10]. In terms of other coherent structures, horseshoe vortices formed by the upstream separated regions due to the adverse pressure gradient caused by the bow shock, wrap around the jet periphery and convect downstream as they are shown in Figure 1 (bottom figure). Counter rotating vortices develop on top of the normal shock (Mach disc) that is caused by sudden expansion of the jet stream. These counter rotating cross flow vortices are assessed by [12] as the primary source of entrainment of surrounding incoming flow air into jet flow that is important for farfield mixing. They are produced by folding of the vortex ring, which is the downstream manifestation of vorticity arising from the sidewall boundary layers of injectors [13]. Finally, wake vortices periodically shed near the base of the inner jet core and trail downstream under the jet plume [12,14].

Gruber *et al*. [7] shed light on the mixing characteristics of *CO*_2_ and Helium transverse jets in Mach 2 cross flow with different injector configurations. Planar Mie scattering images provided global flowfield characteristics, transverse and lateral penetrations for each injector configuration. Instantaneous and time-averaged information concerning the structural organization of the flowfields was obtained. In a similar study by Gruber *et al*. [8] in which air and Helium jets were utilised, it is suggested that injectant molecular weight variations did not strongly affect the penetration of transverse jet into the crossflow, although they led to substantially different compressibility levels that dramatically influence the characteristics of the large-scale structures formed in the shear layer and the entrainment and mixing occurring between the injectant and crossflow fluids. Results also indicated that air jet (low compressibility, based on convective Mach number) contained a larger mixing zone than Helium jet (high compressibility). The relevant parameter of jet penetration is momentum flux ratio, *J*, which is defined below in Equation (1). Recently Schetz and Burger [15] investigated the effect of molecular weight on the transverse injection flowfield with Helium, air, methane injection and yet they found out that the effect of molecular weight is weak.

(1) $$J=\frac{\gamma_{\text{jet}}p_{\text{jet}}M_{\text{jet}}^{2}}{\gamma_{\infty }p_{\infty }M_{\infty }^{2}}$$

where γ is the specific heat ratio, *ρ* is the density, *M* is the Mach number and subscripts ∞ and *jet* refer to freestream and jet conditions respectively.

To understand the flow topology and to extract velocity/mixing information for “jet in high speed flows” is a challenging task experimentally, especially at high cross flow Mach numbers. Small length and time scales associated with high free stream velocities introduce significant challenges in terms of both spatial and temporal resolutions. The presence of low velocity regions coupled with high velocity regions brings further difficulties in terms of dynamic ranges of measurement techniques. In terms of extracting velocity fields only Particle Image Velocimetry (PIV) offers a complete flow diagnostic tool. Capable of providing instantaneous planar velocity fields, PIV has been successfully applied to high Mach number flows in the last decade by Haertig *et al.* [16] and Schrijer *et al.* [17]. Haertig *et al.* [16] applied PIV to nozzle and blunt body flows inside a shock tunnel at Mach 3.5 and 4.5. with freestream velocities more than 1500 m/s. On the other hand, Schrijer *et al.* [17] utilised PIV for a double compression ramp flow inside a Ludwieg tube facility at Mach 7 with a velocity of above 1200 m/s. The extension of PIV to high Mach number regime involves certain challenges such as selection and uniform seeding of appropriate solid particles, the examination of the particle time response and the analysis of particle image recordings where a large variation in particle image density occurs due to shock waves [18].

The present study constitutes a challenging application of PIV to investigate sonic transverse air, *CO*_2_ and Helium jets in Mach 5 cross flow inside a blowdown wind tunnel. It aims to provide good quality reliable experimental data in terms of velocity/turbulence fields and jet penetration, which is not available in the literature. Schlieren visualisation is also conducted to provide a solid foundation in terms of mean flow features as well as inherent unsteadiness.

## 2. Experimental Setup

### 2.1. University of Manchester HSST

**Figure 2.**
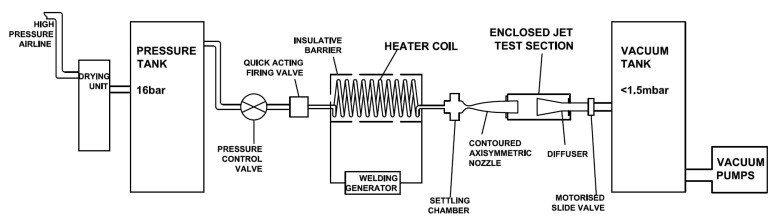
University of Manchester HSST schematic layout by Erdem and Kontis [19].

The experiments are conducted in the High Supersonic Tunnel (HSST) at the University of Manchester. The tunnel is an intermediate blowdown (pressure-vacuum) type which uses dry air as working fluid and is shown schematically in Figure 2. Air from a high pressure airline is dried and stored in a pressure vessel at a pressure over 15 bar. After passing through a pneumatically operated quick acting ball valve, the gas enters the electric resistive heater section. The gas temperature is raised from ambient to a temperature, which is sufficient to avoid liquefaction on its expansion through the nozzle and that of a maximum enthalpy flow condition of 700 K. On leaving the heater, air enters the settling chamber which is downstream of the flow straightener matrix. Immediately downstream of the settling chamber a contoured axisymmetric Mach 5 nozzle is situated. The stagnation pressure can range from 5 to 8 bar and thereby unit Reynolds numbers, *Re/m*, of between 4 – 16 (x000B7) 10^6^ 1/*m* can be achieved [19,20]. The tunnel working section is an enclosed free jet design. The calibration of the facility was carried out by the authors; the variations in Mach number and unit Reynolds number were found to be ±0.4% and ±3.7% respectively [19,20]. The useful running time is found to be 7.5 s. Stagnation pressure *p*_0_, and stagnation temperature, *T*_0_, measurements are done using a Pitot probe attached to an absolute pressure transducer, Kulite XTE-190M (6.89 bar range), and a K-type thermocouple probe at the settling chamber. Analogue signals from the sensors are acquired by a high speed Data Acquisition (DAQ) card, National Instruments (NI) PCI-6251, after they are conditioned by a SXCI-1000 unit. The existing system has the capability of collecting data at a frequency up to 333 kHz at 16 bit digitisation.

### 2.2. Schlieren Visualisation

**Figure 3.**
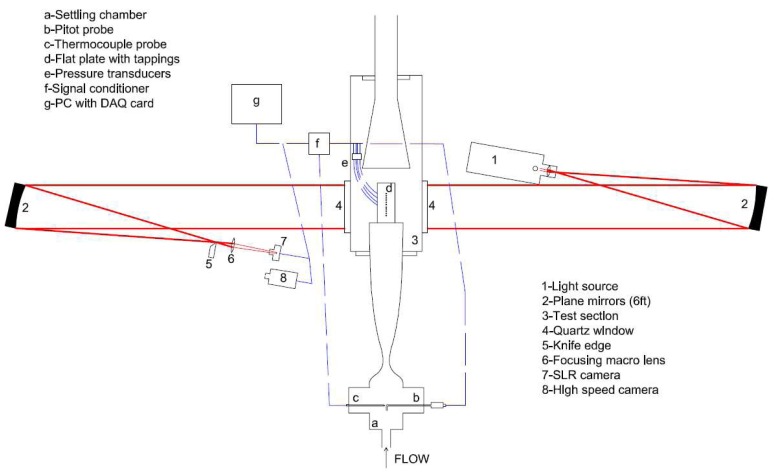
Schematic setup of Schlieren visualisation with data acquisition architecture by Erdem and Kontis [19].

Toepler's z-type schlieren technique, adopted for flow visualisation, consists of a continuous light source of Palflash 501 (Pulse Photonics) with a focusing lens and a 1 mm wide slit, two 203.2 mm parabolic mirrors with 1828.8 mm focal length, a knife edge, a set of Hoya 49 mm close-up lenses and a digital Canon SLR camera, EOS-450D, 12MP A parallel beam of light is passed through the test section windows before focusing on the knife edge plane that is placed perpendicular to flow direction and the focused beam is shone on the CMOS sensor of the camera [19]. The camera is set to continuous recording mode at 3.5 fps with full resolution; the shutter speed is adjusted to maximum value of 1/4000 s with an ISO speed of 400 to provide enough detail and appropriate brightness. The digital resolution is approximately 34 pixels per mm. In addition a high speed Photron SA-1 High Speed Video system is utilised to record time-resolved Schlieren images up to 675,000 fps at various pixel resolutions and shutter speeds. The optimum frame rate is based on a compromise between adequate temporal resolution and pixel resolution. The shutter speed is set to 1 *μ*s to resolve flow features with sharpness. The digital resolution is approximately 10 pixels per mm. The layout of the optical setup and the data acquisition architecture with measurement chain is shown in Figure 3.

### 2.3. Test Model

**Figure 4.**
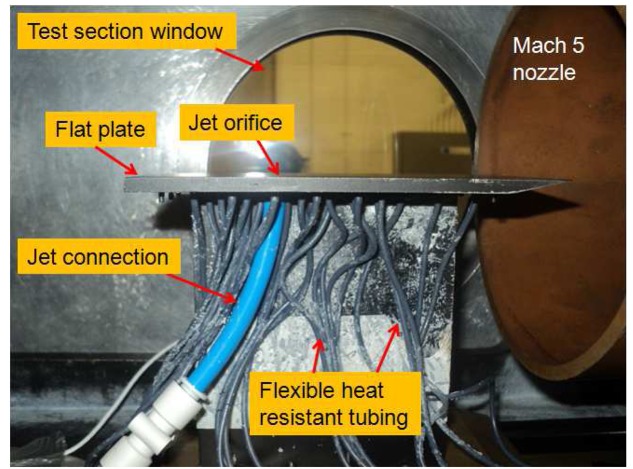
Flat plate model placed inside test section.

The model used for this study is a sharp leading edged flat plate (leading edge thickness smaller than 100 *μm*) with a converging circular jet orifice of 2.2 mm in diameter, through which a sonic turbulent jet of air, *CO*_2_ and Helium jets are injected after regulation. The flat plate is 155 mm long, 68 mm wide and 5 mm thick and painted matt black to avoid reflections for PIV application. The jet orifice is located at 105 mm from the leading edge at the centreline. The jet stagnation pressure, *p*_0_*_jet_,* is adjusted with the help of a pressure transducer, Kulite XTE-190M (3.5bar range) that is connected to an 8 mm air pipe via T-junction. The model, situated inside the test section, is shown in Figure 4.

### 2.4. PIV Measurement Technique

Two component PIV measurements are carried out on the centreline of the model with a dedicated PIV system, which includes a seeding device that discharge particles through the jet orifice, an illuminating laser with related optics to create a laser sheet and a recording camera. The following subsections describe the subsystems; these can also be found in [21].

#### 2.4.1. Seeding

**Figure 5.**
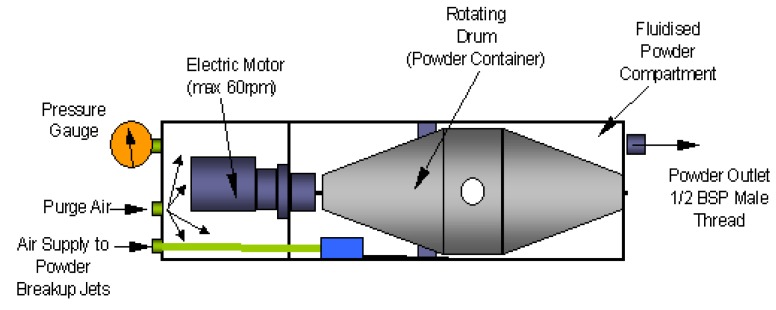
Schematic of PS-10 powder seeder device.

The particles seeded into flow field through the jet orifice, enable to visualize and then to extract the velocity information of the fluid motion. As a standard requirement, they should follow the flow in a coherent homogeneous way with minimum velocity lag and at the same time they should provide sufficient illumination to be recorded with adequate contrast. Furthermore they have to withstand the free stream conditions of the HSST working section. For this purpose aluminium oxide powder particles (dehydrated prior to experiments) with a nominal crystal size of 300 nm are used with a nominal bulk density of *ρ_p_* = 3970 *kg/m*^3^. A PS-10 powder seeder device from Dantec Dynamics, is used to generate an airflow seeded with particles. This device consists of a rotating drum that is controlled by an electronic motor inside a pressure vessel. The drum that contains powder is rotated about a horizontal axis; each revolution of the drum ensures a small amount of powder is dispensed through a small opening. Inside the chamber there are six sonic break-up jets to prevent agglomeration of the particles. Baffles that are attached to outer perimeter of the drum also help to agitate heavy agglomerates. In order to prevent powder particles leaking back to the upstream half of the chamber and contaminating the region where the electric motor is housed, purge air is continuously supplied. A single exit port of 10 mm is located on the side of the chamber to allow seeded airstream flow towards the rig via an air tube. When the drum is not rotated the seeder ceases to dispense particles thus the chamber acts solely as a pressurised vessel, that can be controlled via pressure transducers. A schematic is shown in Figure 5.

The flow tracing capability of particles of diameter, *d_p_,* and a particle density, *ρ_p_,* with a fluid viscosity, *μ_f_,* is usually quantified through the particle relaxation time, *τ_p_.* The theoretical behaviour for small spherical particles may be reduced to the modified Stokes drag law defined by Melling [22]. Given the relatively low value of the Mach number and Reynolds number based on the particle diameter, the modified drag relation that takes into account rarefaction effects yields the expression for the relaxation time in Equation (2), where *Kn_d_* is the Knudsen number based on the particle diameter, which is defined in Equation (3). *Re_d_* is the Reynolds number based on the diameter of the particle and *M_υ_* is the Mach number both evaluated for the maximum particle slip velocity *ΔV,* [18].

(2) $$\tau_{p}=\frac{\rho_{p}d_{p}^{2}(1+2.7Kn_{d})}{18\mu_{f}}$$

where

(3) $$Kn_{d}=1.26\sqrt{\gamma }(M_{\upsilon }/{\text{Re}}_{d})$$

As suggested by Samimy and Lele [23], the particle dynamic effects may be further quantified by the Stokes number, *Sk,* written in Equation (4). For accurate flow tracking the time scale of the flow has to be greater than the time response of the particles, *i.e., Sk* ≪ 1. The characteristic jet flow time scale of can be found by assuming Δ*V* as *U_jet_* and *δ* (shear layer thickness) as *d_jet_* (see Equation (4)), whereas the particle time response can be calculated using sonic jet conditions for air, *CO*_2_ and Helium gases (*T*_0*jet*_ = 295 *K, U_jet_* = 315, 250, 875 *m/s* respectively). The respective timescales and Stokes numbers are tabulated in Table 1. The flow following capability of aluminum oxide particles for Helium injection is the most critical one (with a *Sk* of 0.14) due to highest jet velocity and biggest particle relaxation time; whereas for air and *CO*_2_*, Sk* ≪ 1 condition is clearly satisfied.

(4) $$\begin{array}{cc} Sk=\frac{\tau_{p}}{\tau_{f}}\text{where} & \tau_{f}=10\frac{\delta }{\Delta V}\approx 10\frac{d_{\text{jet}}}{U_{\text{jet}}} \end{array}$$

#### 2.4.2. Illumination

A Litron Nano L series, Nd:Yag Q-switched laser is used for PIV illumination. The laser has the pulse energy of 200 mJ at repetition rate of 15 Hz. The laser beams are pulsed at the wavelength of 532 nm. The pulse width of the light is 6 ns and the pulse separation time (the time interval between two consecutive PIV images light pulses, Δ*t*) can be adjusted to 0.1 *μ*s as minimum. A laser sheet of 0.5 mm thickness is produced with a series of spherical and cylindrical lenses and directed above the test section via a laser guided arm.

#### 2.4.3. Image Recording

A LaVision Imager ProX2M CCD camera with 1600 × 1200 *pixel*^2^ resolution (with 7.4 *μm* pixel pitch) is used to record scattered light reflecting from particles at 14 bit digitisation. The camera is equipped with a Sigma 105 mm focal objective lens with *f* number of 5.6, in combination with a narrow-bandpass 532 nm filter in order to minimize ambient light interference. The whole operation is synchronised and run using DaVis 7.2 software with a Programmable Timing Unit (PTU).

#### 2.4.4. Setup

**Figure 6.**
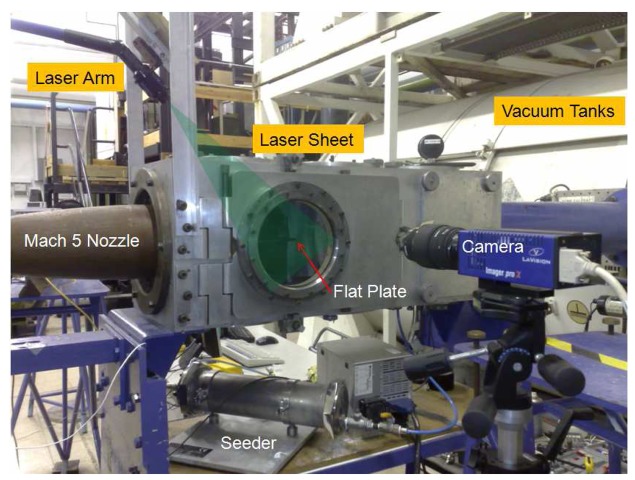
PIV setup.

The PIV setup is arranged such that it can produce a laser sheet that is tilted at 45 degrees with respect to the flat plate, as it is shown in Figure 6. The laser sheet is localised at the centreplane where the flow field can be safely assumed symmetrical with respect to the centreplane. However, the laser sheet thickness of 0.5 mm compared to 2.2 mm jet diameter introduce some effects of unavoidable out of plane motion into measurements. In the tests, only the transverse jet is seeded with aluminium oxide powder particles and the jet stagnation pressure is measured on the line with a Kulite XTE-190M (3.5 bar range) pressure transducer just before the jet orifice, prior to experiments with the seeder drum being idle. During the test run the pressure transducer is removed to avoid deposition of particles inside the diaphragm of the sensor and the seeding density level remains stable. Pulse separation between laser pulses, Δt of 0.6 *μ*s for air and *CO*_2_ jets and 0.3 *μ*s for Helium jet are set so that sufficient displacement for the tracer particles of between 4.5 to 10 pixels for the velocity range from 250 m/s to 875 m/s can be achieved.

The camera sees a Field of View (FoV) orthogonally to the laser sheet. The flowfield is imaged in the streamwise (x) and wall-normal (y) directions over a FoV of approximately 64 × 48 *mm*^2^, resulting in a digital resolution of approximately 25 pixels per mm. A dataset of around 100 instantaneous vector fields is acquired during the test time at 15 Hz. Recorded images are divided into initial interrogation areas (IAs) and then processed with a cross correlation algorithm using DaVis 7.2 software. The initial interrogation areas are selected as 32 × 32 *pixel*^2^ with 2 passes and then refined to 16 × 16 *pixel*^2^ with 3 passes. A 75% overlap is employed in order to improve spatial resolution. A median filter is applied to correct for spurious vectors. The median filter computes a median vector from 8 neighbouring vectors and compares the middle vector with this median vector ± deviation of the neighbouring vectors. The centre vector is rejected when it is outside the allowed range of the average vector ± deviation of the neighbouring vectors. This rejected vector is iteratively replaced using a 4-pass regional median filter. This filter is ideally suited whenever it is required that the vector field should not contain any spurious vectors, even with the drawback that some good vectors are rejected. It is essential that it is applied when calculating averages and standard deviations [24]. FoV averaged signal to noise ratio (SNR, the ratio of the first correlation peak to the second peak) is found to be better than 2.6, which is deemed to be very good quality [24]. Vector validation scheme discards any vector with a SNR value under 1.8. In terms of peak locking phenomenon (when seeding particles are too small and produce particle images on the CCD of less than one pixel in diameter) the relevant peak locking parameter defined in DaVis 7.2 Manual [24] is found to be around 0.15 implying very weak peak locking towards integer values of displacement. This outcome may be due to the agglomerated particle behaviour forming a bigger image on the chip of the camera rather than the nominal particle size of 300 nm. Nevertheless the agglomeration has consequences on particle response time.

## 3. Results

### 3.1. Upstream Conditions

Different experimental conditions have been studied corresponding to three different gases with same momentum flux ratio, *J*. The flow conditions for the tests are tabulated in Table 2 together with the associated experimental uncertainties found using an approach from Moffat [25]. These conditions are deduced from stagnation pressure (*p*_0_, *p*_0_*_jet_*) and stagnation temperature (*T*_0_) signals. Useful flow time is reached 0.6 s after the flow starts. *p*_0_ signal for HSST varies less than 1% from 0.6 s to 7.8 s whereas *p*_0_*_jet_* signals vary less than 0.1% throughout the measurement period.

### 3.2. Conventional/High Speed Schlieren Photography

**Figure 7.**
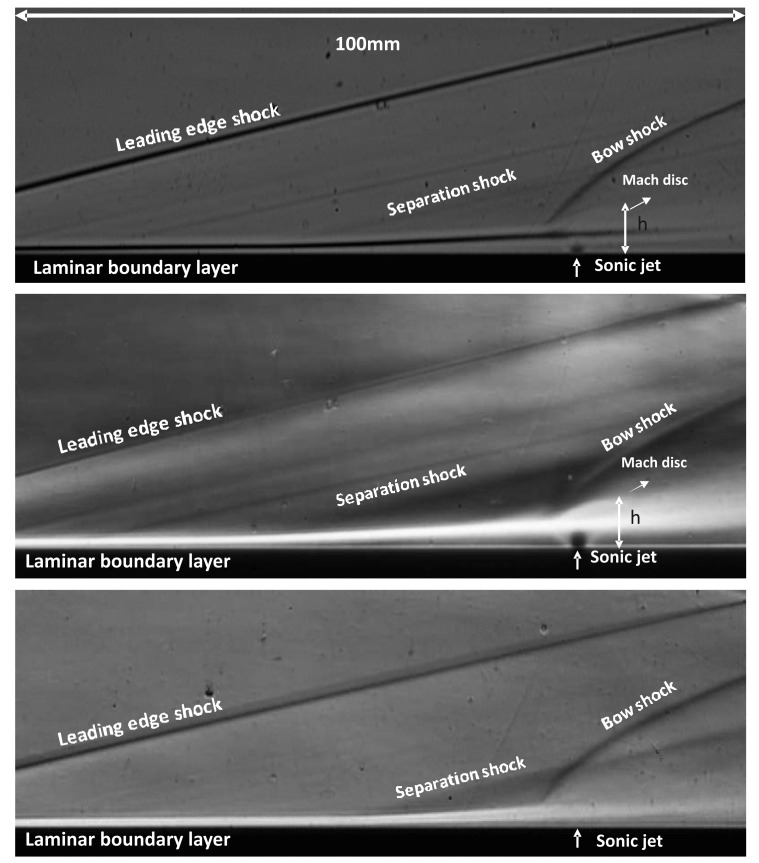
Schlieren visualisation of the flowfield in the presence of the sonic transverse jet with annotated flow structures; **top**: air, **middle**: *CO*_2_, **bottom**: Helium.

Figure 7 shows the long exposure (250 *μ*s) schlieren images of the flowfield for all the gases as shown in Table 2. A leading edge shock due to viscous interaction at the leading edge of the plate and a laminar boundary layer growing up to the separation point and then deviating towards vertical direction thereafter can be observed clearly. The high speed boundary layer developing on the flat plate before the jet induced separation within the achievable range of unit Reynolds numbers, is laminar unless tripped [19]. Whereas the jet flow is turbulent for the range of Reynolds numbers (see Table 2). Due to the separation of the incoming laminar boundary layer, transition to turbulence is likely to occur within the upstream separation regions. The cause of this phenomenon is the extreme sensitivity of the separated shear layer to disturbances [26]. Separation shock emanates around the separation point and intersects the jet induced bow shock; the sonic jet expands suddenly and bends downstream, afterwards its expansion is terminated by the Mach disc. The maximum vertical position of the Mach disc is taken as the Mach disc height, *h.* The nearfield boundary of the jet is confined within the barrel shocks. The separation region, separation shock and bow shock are three dimensional curved flow structures around the transverse jet. These three dimensional flow structures are superimposed on schlieren images and are well reported in the literature and mentioned in Section 1. The separation region is clearly different for different gases; *CO*_2_ jet produces a bigger separation region and Helium jet has the smallest region. This phenomenon can be interpreted as an effect of the molecular weight based on high sonic velocity at the jet orifice (about 875 m/s for Helium compared to 315 m/s for air at Mach 1). Higher jet velocities usually translate into high convective Mach numbers, which imply reduced mixing [27]. This phenomenon may cause reduced blockage of the jet flow. In terms of bow shock position all the jets have similar bow shock pattern suggesting similar penetration structure.

The Mach disc height, *h,* can be extracted from the schlieren images using digital image processing. The Mach disc height is compared to a theoretical estimate from Cassell [28], which is shown in Equation (5). *C_d_* is the discharge coefficient of the sonic jet with values around 0.96–0.98 for the range of jet Reynolds numbers considered. These values are tabulated in Table 3. The agreement is found to be good since jet penetration height is governed heavily by *J*. In addition, the stagnation conditions for both the jet and the free stream are quite steady (less than 1% variation) during the useful running time of the HSST and they are known accurately.

(5) $$\frac{h_{\text{theo}}}{d_{\text{jet}}}=\frac{1}{M_{\infty }}\sqrt{\frac{2p_{0\text{jet}}\gamma_{\text{jet}}}{C_{d}p_{\infty }\gamma_{\infty }}}G$$

where

$$G={\left[\frac{2}{\gamma_{\text{jet}}-1}{\frac{2}{\gamma_{\text{jet}}+1}}^{\left(\gamma_{\text{jet}}+1/\gamma_{\text{jet}}-1\right)}\left\{1-{\frac{p_{\infty }}{p_{0\text{jet}}}}^{\left(\gamma_{\text{jet}}-1/\gamma_{\text{jet}}\right)}\right\}\right]}^{1/4}$$

**Figure 8.**
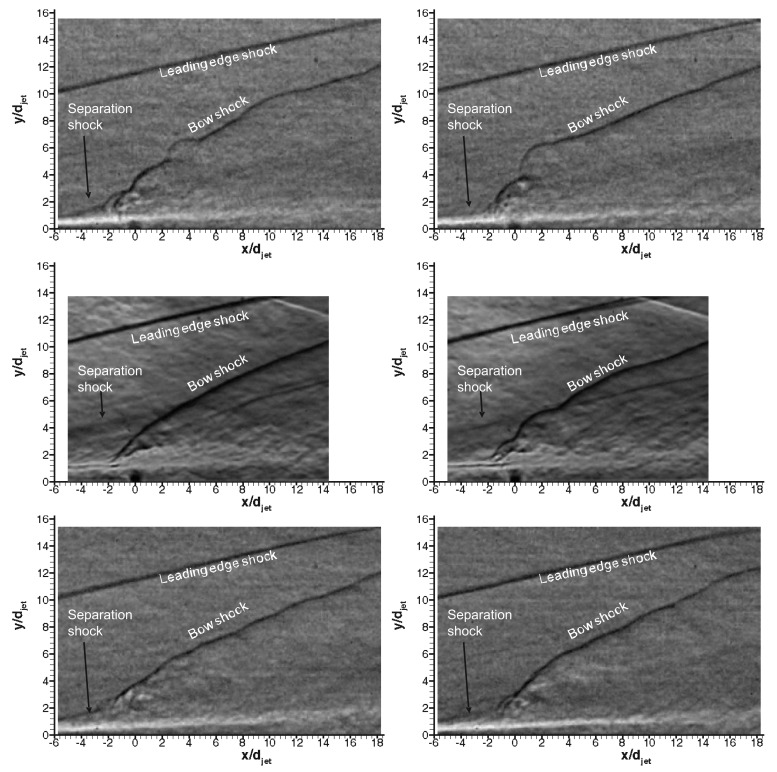
Two instantaneous schlieren images with annotated flow structures; **top**: air, **middle**: *CO*_2_, **bottom**: Helium.

As mentioned in Section 1, the interaction of the transverse jet with the incoming flow is unsteady owing to jet shear layer instabilities. In the region near the injector exit, the injectant fluid moves with a higher vertical velocity tangentially to the interface than the incoming flow. As a result, large vortices are periodically formed engulfing large quantities of free stream fluid and drawing it into the jet shear layer and then are convected downstream. These large scale coherent structures are dominant in the jet shear layer and their structural evolution might have a big influence on the jet near field [10]. It is therefore important to understand how these structures and their growth rates change in time. High speed schlieren photography reveals these structures captured at 16 kfps with 1 *μ*s exposure as they are shown in Figure 8 for three gases. Several interesting features, such as the large scale structures at the jet periphery and the distorted bow shock are apparent in the images. The bow shock stand-off distance is very small, it is almost merged within the expanding jet, and curves sharply downstream. The local shape of the bow shock appears to depend strongly on the convection of large scale shear layer structures, especially close to the jet exit where local flow behind the bow shock is subsonic. Furthermore the separation shock is also unsteady due to the disturbances, in the vicinity of the jet injection that are fed upstream through the boundary layer. The barrel shock and the Mach disc are, however, not very clear in the short exposure schlieren images, due to the unsteadiness. The shear layer eddies are part of the unsteady Kelvin-Helmholtz spanwise rollers wrapping around the jet. They are the traces of three dimensional transverse vortex tubes whose cores coiled up around the jet with their legs connected downstream of the jet exit [10]. In terms of penetration characteristics all jets exhibit similar penetration pattern.

**Figure 9.**
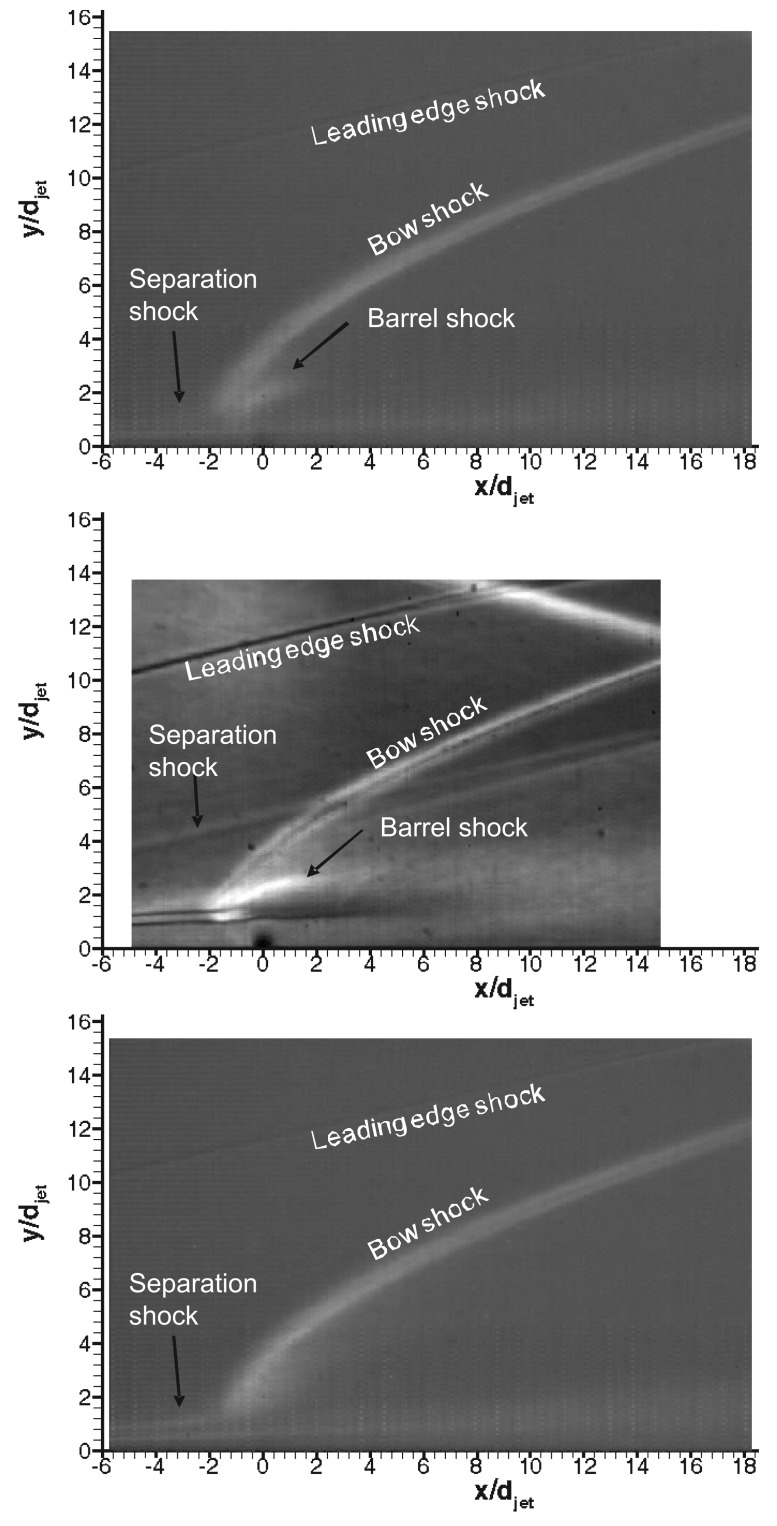
RMS of 1000 instantaneous schlieren images with 3 gases; **top**: air, **middle**: *CO*_2_, **bottom**: Helium.

Root Mean Square (RMS) of the fluctuations in the light intensity based on 1000 schlieren images, which show different levels of penetration and signify the high levels of unsteadiness, are shown in Figure 9 for each gas. The jet upper boundary can be easily seen and high amplitudes of RMS are observed to occur in the flow domain occupied by the fluctuating bow shock and the windward side (upstream side) of the barrel shocks. It has to be noted that the evolution of coherent jet shear layer vortical structures cannot be discussed here because of the long interframe time of the schlieren recording, which is 62.5 *μs*. The leading edge shock is observed as a very thin line (the unsteadiness is minimal), which demonstrates the good flow quality at upstream conditions.

### 3.3. PIV

**Figure 10.**
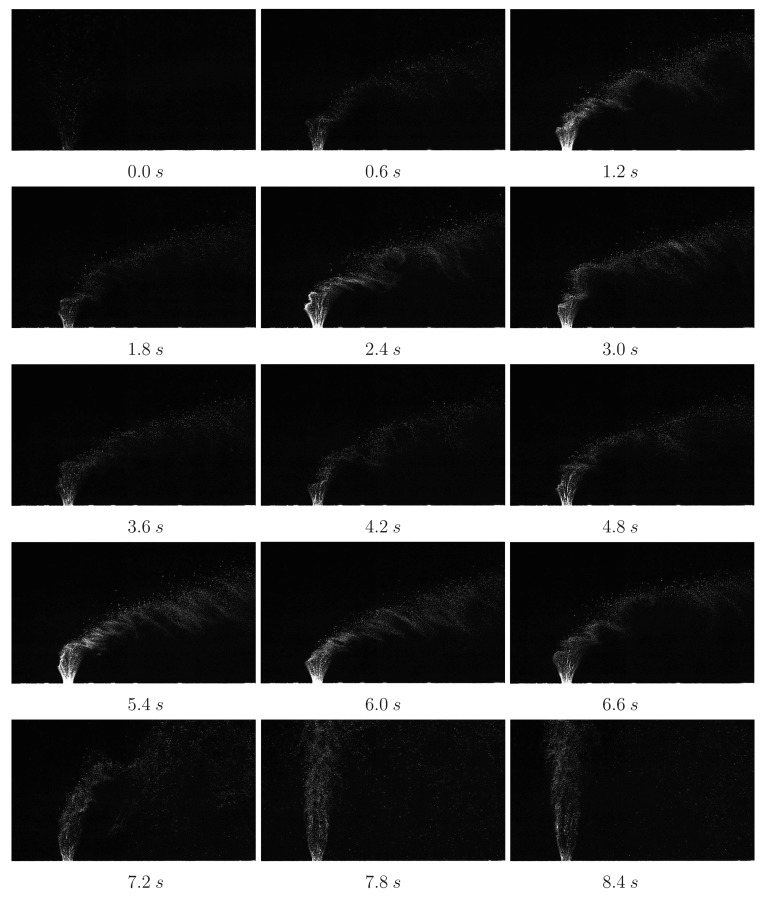
PIV raw image visualisation of the sonic transverse air jet captured at 15fps during the running time of HSST. Only the first frames of the PIV recordings are shown.

Figure 10 shows the raw PIV images captured throughout the test run for air jet. In the experimental test campaign the transverse jet is started just before the test gas arrival. In that time the jet discharges nearly vertically (minimum effect of the vacuum downstream) due to significant initial jet momentum flux ratio. Afterwards with the arrival of the freestream test gas, transverse jet bends towards the main direction of the freestream flow, in the horizontal direction, x. Unsteadiness of the jet trajectory and jet shear layer instabilities are observed clearly. Periodically formed large vortices engulf large quantities of incoming air, drawing it into the jet shear layer, and then are swept downstream. After the useful running time has passed (around 7.2 s), severe oscillations start to occur in the jet flow as Mach 5 flow no longer exists and finally when the firing valve is closed (when there is no cross flow) the jet discharges nearly vertically. The early termination of useful running time (7.2 s instead of 7.5 s) is attributed to the fact that a constant mass flow rate of the secondary jet is increasing the mass flow rate going inside the vacuum tanks, and hence increasing the back pressure.

**Figure 11.**
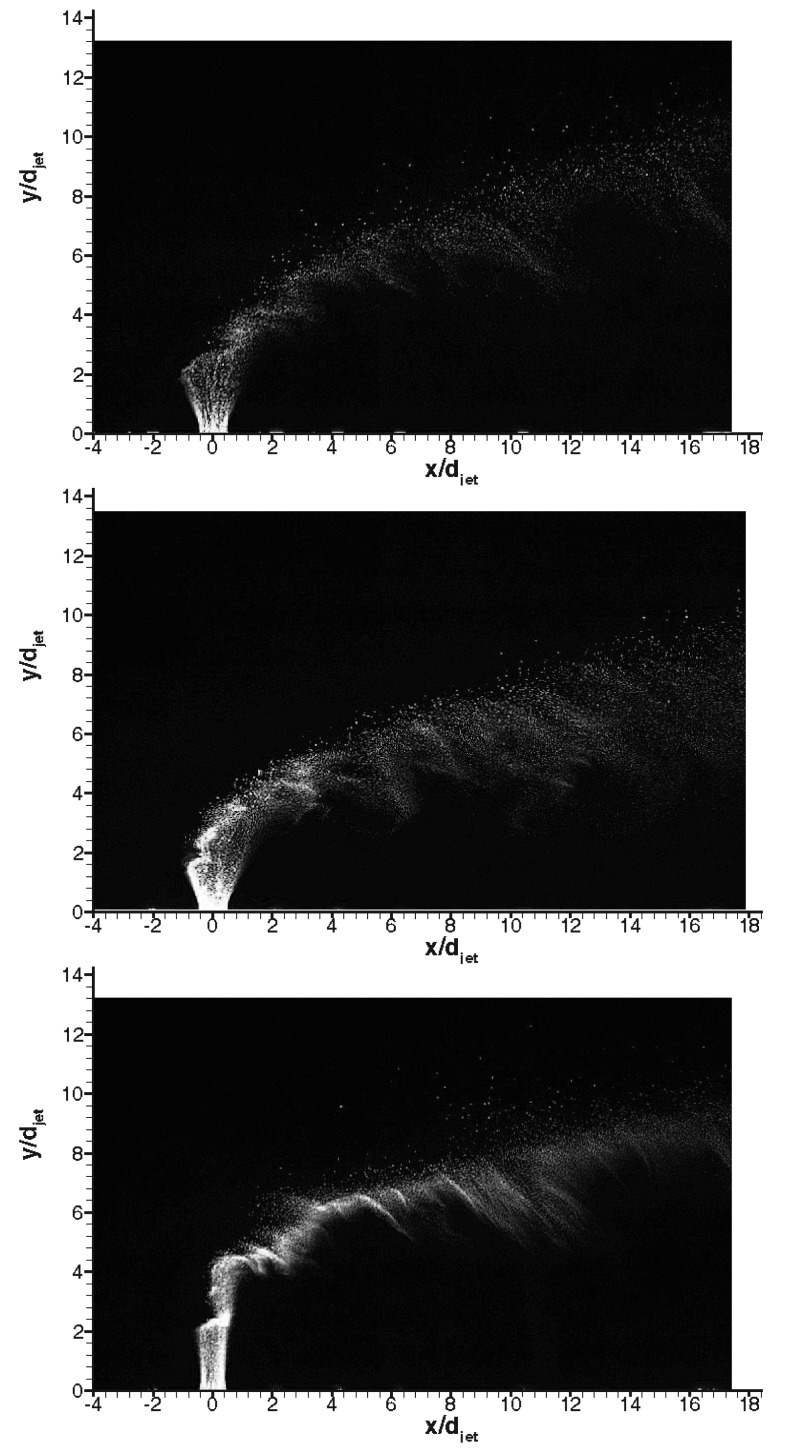
Representative instantaneous PIV raw images; **top**: air, **middle**: *CO*_2_, **bottom**: Helium.

**Figure 12.**
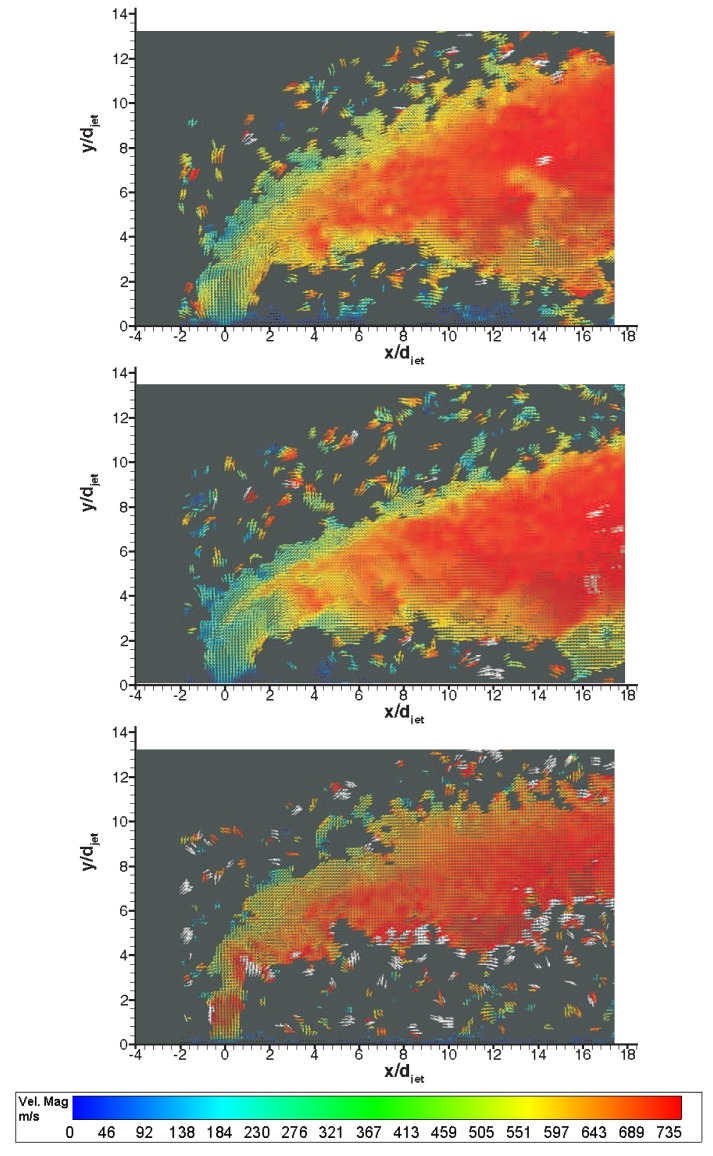
Representative instantaneous velocity vectors; **top**: air, **middle**: *CO*_2_, **bottom**: Helium.

Figure 11 shows instantaneous raw PIV images with different gases in Mach 5 cross flow signifying the difference in penetration characteristics. The penetration trajectory is clearly affected by type of the gas in both nearfield and farfield. In the nearfield air and *CO*_2_ (despite being heavier than air) jets behaves similar in terms of initial expansion of the jet, however Helium jet expands little, discharges on vertical direction for about 3 jet diameters and then bends towards the main stream. This is believed to be because of high jet velocity and hence high convective Mach number. In terms of farfield structure Helium spreads minimal compared to others whereas *CO*_2_ jet convects closer to wall compared to air jet. Figure 12 shows corresponding velocity vector fields of the raw PIV images with different gases. The velocity vectors are coloured by velocity magnitude, *i.e.,* $\sqrt{u^{2}+\upsilon^{2}}$, where *u* and *υ* are stream wise and transverse velocity components respectively. White regions specify velocities above 750 m/s. The main flow is in the x direction and the jet orifice is located at the origin. The horizontal and vertical coordinates are normalized with the jet diameter, *d_jet_*. The time that elapses between consecutive recordings of 15 Hz is significantly larger than the jet flow characteristic time scale is between 25–88 *μ*s, leading to the measurement of uncorrelated velocity fields. With the current spatial resolution (two adjacent velocity vectors are separated by approximately 0.2 mm) it is inherently implied that global dynamics of the jet crossflow interaction is deduced. As the jet is discharged from the orifice, at a velocity around 315 m/s and 250 m/s for air and *CO*_2_ jet respectively, acceleration of the flow in the transverse direction is observed and terminated by the Mach disc which bends the jet towards cross flow. After the normal shock, the jet velocity is reduced followed by an acceleration reaching values of 750 m/s, which is close to the free stream velocity but slightly lower due to the presence of the bow shock. On the other hand Helium jet experiences deceleration after exiting the jet orifice and bends towards direction of the crossing stream sharply.

**Figure 13.**
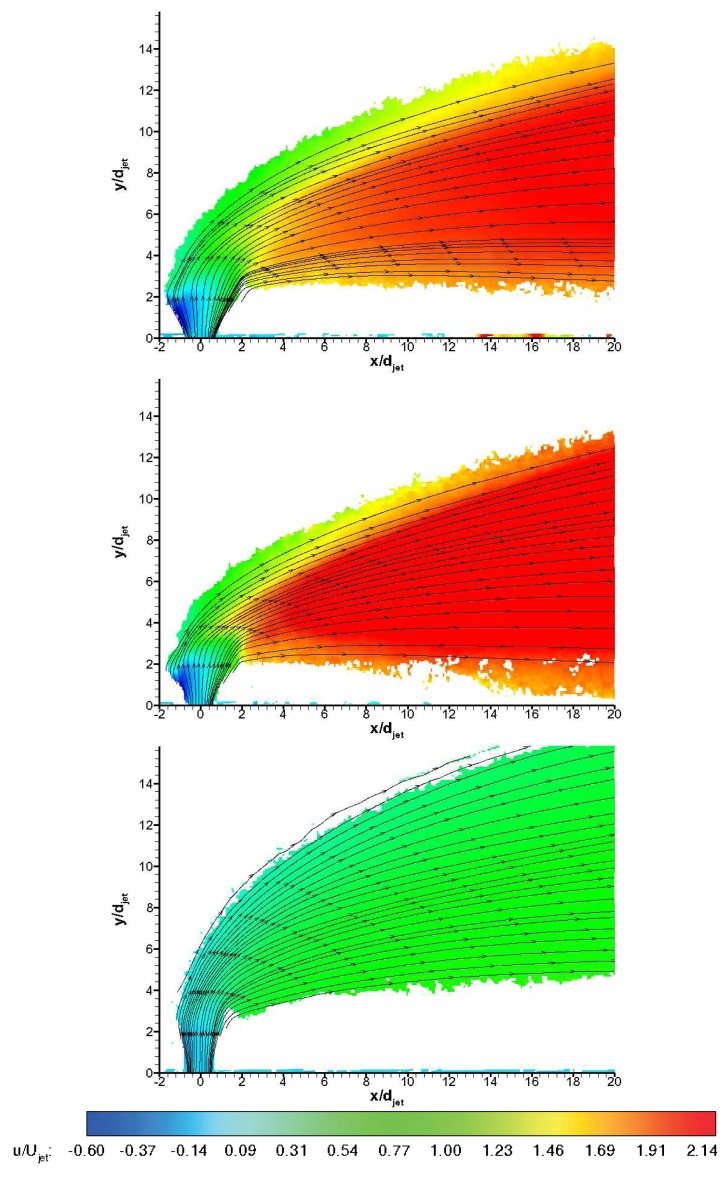
Mean streamwise velocity *u/U_jet_* contours with pathlines; **top**: air, **middle**: *CO*_2_, **bottom**: Helium.

**Figure 14.**
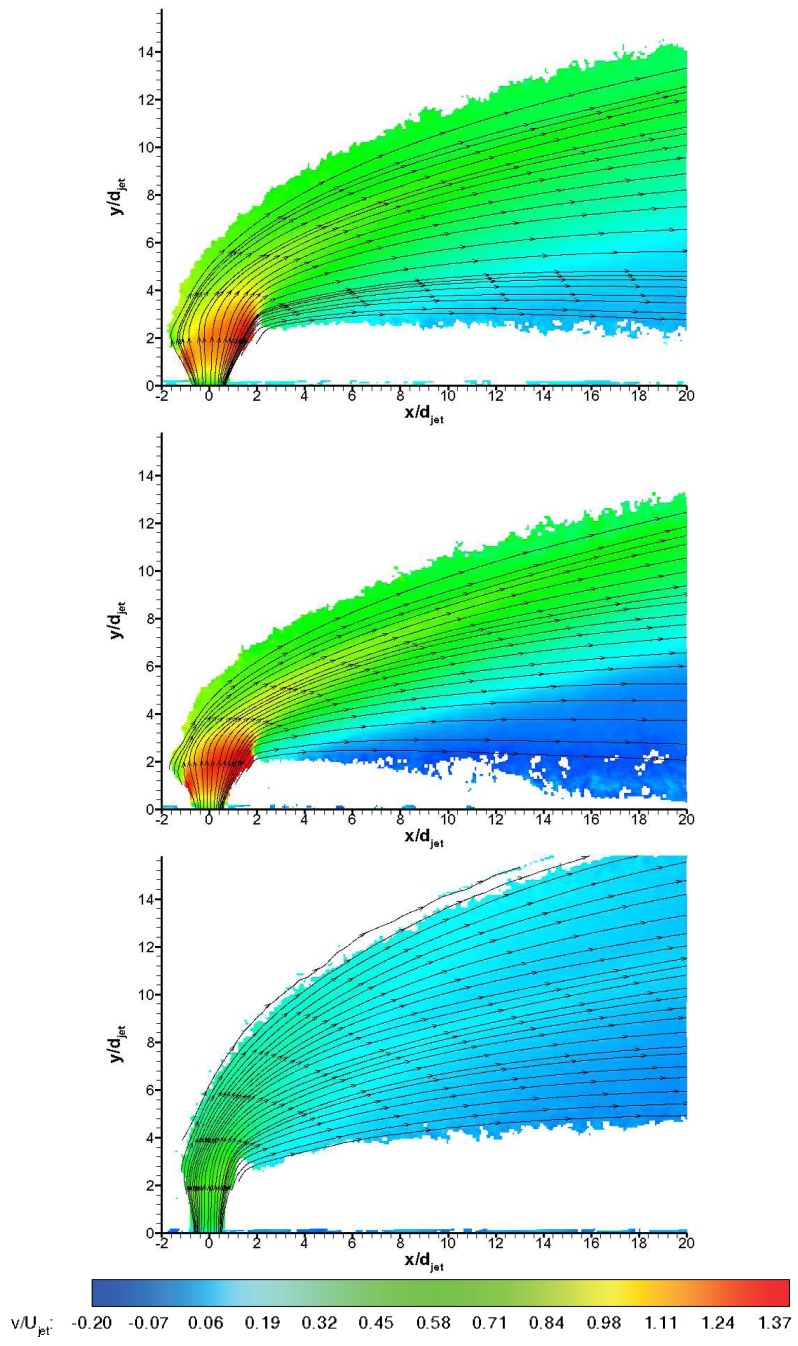
Mean transverse velocity *υ/U_jet_* contours with pathlines; **top**: air, **middle**: *CO*_2_, **bottom**: Helium.

Figures 13 and 14 show non-dimensionalised streamwise and transverse velocity contours, *i.e.,u/U_jet_* and *υ/U_jet_,* for three gases ensemble-averaged over 100 instantaneous vector fields during the useful running time of HSST Pathlines are also visualised. The unsteady jet shear layer structures do not appear in averaged velocity contours naturally. Leaving the jet orifice, the transverse jet expands depending on the back pressure behind the jet induced bow shock. On the windward side (upstream side), the jet turns quickly towards the main flow direction, whereas on the leeward side (downstream side) turning behaviour is more gradual. On the leeward side the termination of the jet expansion (Mach disc) is especially apparent for air and *CO*_2_ jets. Negative streamwise velocities are observed on the windward side of the jet stream in Figure 13, whereas negative transverse velocities are observed in the farfield on the bottom region in Figure 14. Air jet shows medium penetration path and expands substantially in farfield, whereas *CO*_2_ jet convects closer to the wall after a tight turn to main flow direction. On the other hand Helium jet bends abruptly after considerable vertical penetration and convects far from the wall. All of the jets reach nearly values below freestream velocity at the downstream regions, *i.e.,* after *x/d_jet_* of 8 (for Helium jet there is deceleration to main flow velocity) where they are carried by the main flow behind the jet induced bow shock wave. Penetration behaviour is clearly different with different injectant gases, which is in line with the findings in the literature [10,15]. Due to the low levels of seeding outside the jet boundaries the data is likely to include some level of bias towards to the velocity values in the regions with sufficient seeding density. This velocity bias is most likely to tend to approach to the transverse velocity component in the jet nearfield whereas in the farfield it is expected to tend to approach towards the streamwise velocity component. Therefore the data outside the pathlines is questionable in absolute terms. The uncertainty in the ensemble-averaged mean velocities based on jet velocity is tabulated in Table 4, using an approach from Humble [29].

To assess the particle response time experimentally, an oblique shock wave test [16,18] or a blunt body normal shock wave test [30] are conducted. However the lack of a standing oblique shock for this flowfield suggests the use of a Mach disc (a normal shock) instead. Even though the acceleration of the flow before and after the Mach disc introduces uncertainty in terms of the velocities before and after shock, the possibility of the ensemble averaging during the useful running time of HSST diminishes the amount of uncertainty in ensemble-averaged flow field. This is due to the fact that the uncertainty in mean velocity is inversely proportional to the square root of the ensemble size. Ragni *et al.* [31] described two critical parameters, as spatial ratio, *SR,* and temporal ratio, *TR,* for the experimental measurement of the particle response time. The expressions for these parameters are specified in Equation (6). *IA* is the size of the final interrogation area and ξ*_p_* is the relaxation distance.

(6) $$\begin{array}{ccc} SR=\frac{IA}{\xi_{p}} & and & TR=\frac{\Delta t}{\tau_{p}} \end{array}$$

**Figure 15.**
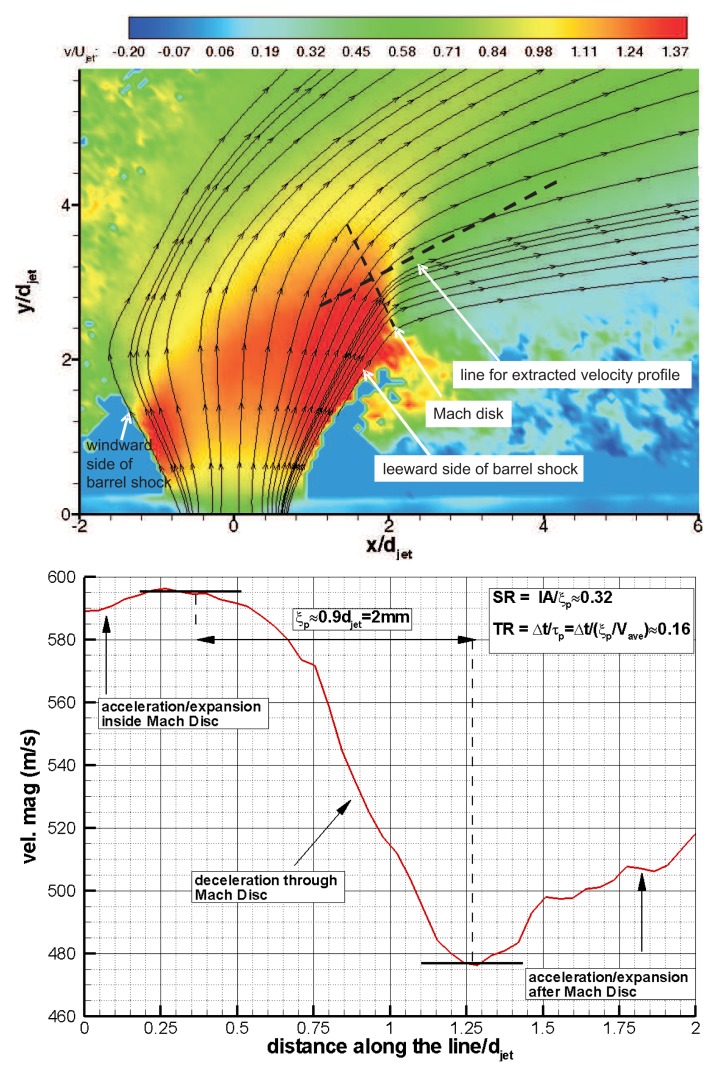
**Top**: mean transverse velocity *υ/U_jet_* contours with pathlines around the air jet orifice; **bottom**: mean velocity magnitude profile extracted through the Mach disc for particle response assessment.

For a PIV experiment, the conditions for *SR, TR* ≤ 1 can be considered acceptable for an accurate measurement of the tracer response time [31]. This condition has implications on the required seeding concentration and digital imaging resolution as well as on the time separation between exposures. In the current experimental campaign, the *SR* and *TR* values are found after measuring ξ*_p_* and *τ_p_* values from the ensemble-averaged flow field for air jet through Mach disc. As there is significant deceleration through the Mach disc, particles cannot adjust themselves as quickly as the jet flow, which causes blurring. In addition the unsteady shock motion in the Mach disc also causes blurring for each instantaneous velocity vector field. When instantaneous vector fields are ensemble-averaged to obtain an averaged flow field, a combined effect of blurring is exhibited around the Mach disc. To find the experimental relaxation distance and response time of the particles, a velocity profile normal to Mach disc is extracted for air jet and plotted in Figure 15. *ξ_p_* and *τ_p_* values are found to be 2.0 mm and 3.7 *μ*s respectively and the criteria of *SR, TR* ≤ 1 for the assessment of particle response behaviour are satisfied.

**Figure 16.**
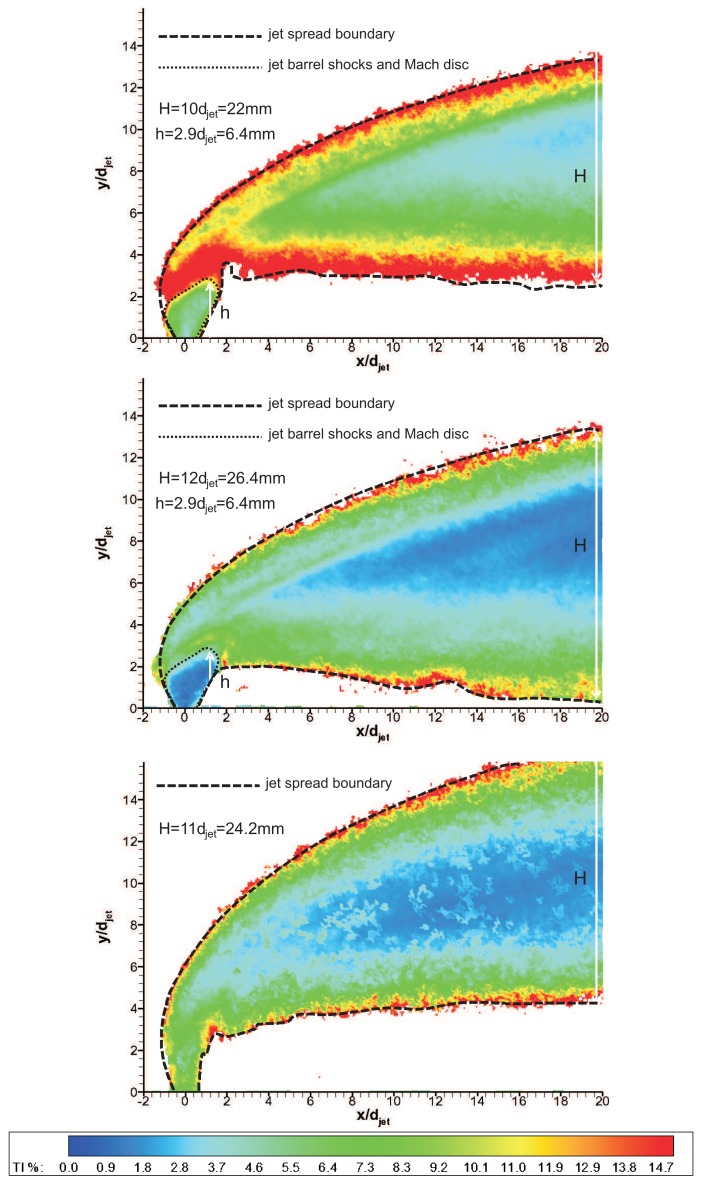
Turbulence intensity (TI) contours; **top**: air; **middle**: *CO*_2_; **bottom**: Helium.

Figure 16 shows the sum of RMS of streamwise and transverse fluctuations ( $\sqrt{\bar{u^{\prime 2}+\upsilon^{\prime 2}}}/U_{\text{jet}}$ *, i.e.,* turbulence intensity, *TI*) contours over 100 instantaneous vector fields for three gases during the useful running time of HSST. These results reflect the interaction that takes place within the flowfield between the jet with the freestream flow and the distributed nature of the turbulence. Standard deviation images produce information regarding the large-scale mixing/entrainment and reveal the mixing zones [9]. Therefore the jet boundaries and the associated penetration characteristics can be demonstrated using RMS contours. The barrel shocks and Mach disc forming the initial jet boundary could be clearly seen and were visualised/quantified for the first time in literature using PIV by [21]. The upper boundary of the jet spreading is defined by the maximum penetration of the shear layer vortices. On the other hand the lower boundary of the jet spreading is defined by the jet stream above the wake vortices that are mentioned in Section 1. Therefore the penetration bandwidth, *H*, can be related to the difference between the extents of the jet boundary. The biggest penetration band is observed for *CO*_2_ jet at 20 jet diameters downstream, on the other hand Helium jet expands significantly at downstream locations. Maximum turbulence occurred above the Mach disc due to the presence of the shear layer and at the intersection of the windward side of the barrel shock and bow shock. Substantial increase in TI occurs outside jet spreading boundaries, leading to non-physical turbulence information (an artefact of PIV image correlation in the absence of necessary particle population). Therefore a TI threshold value is applied to discard these regions, which is taken as 20%.

Mach disc was clearly apparent with low turbulence region around the jet vicinity and its shape was similar for air and *CO*_2_ jets. For Helium jet, Mach disc cannot be observed like in schlieren images and the turbulence level was found to be higher at the jet core due to higher jet velocity However in the farfield, the turbulence intensity level is smaller compared to other gases in the core. Mach disc height values are in very good agreement with the values found from schlieren images as well as the theoretical estimates from Equation (5) (see Table 3). Penetration bands, *H,* at *x/d_jet_* = 20 are also tabulated. However an important point to note is that the uncertainty in *h* is found to be ±0.5 mm for Mach disc height and ±1 mm for penetration band in PIV measurements due to the finite response time of particles. The uncertainty in the ensemble-averaged RMS velocities based on jet velocity is tabulated in Table 4, using an approach by Humble [29].

#### Uncertainty Estimates

The statistical errors in ensemble-averaged mean velocity and turbulence intensity are depicted in Equations (7) and (8). Another source of error is found in the determination of pixel displacement by cross correlation algorithm, *E_cc_*. It is commonly assumed as one tenth of a pixel [32] and is converted to velocity as 6.7 m/s using digital resolution of 25 pixels per mm and Δ*t* of 0.6 *μ*s for Air and *CO*_2_ jets and 13.4 m/s for *He* jet. All uncertainty values are normalised by jet velocity, *U_jet_,* and tabulated in Table 4.

(7) $$E_{<U>}=\sqrt{<u^{\prime 2}+\upsilon^{\prime 2}>}/\sqrt{m}$$

(8) $$E_{\sqrt{<U^{\prime 2}>}}=\sqrt{<u^{\prime 2}+\upsilon^{\prime 2}>}/\sqrt{2m}$$

where *m* is the number of uncorrelated individual vector fields, which is 100.

## 4. Conclusions

Transverse air, *CO*_2_ and Helium injection into Mach 5 crossflow at a unit Reynolds number of 13×10^6^ 1/*m* was investigated experimentally to study the effect of injectant gas on jet interaction flowfield. Sonic turbulent jet was injected through a circular orifice situated at the centreline of a flat plate with a sharp leading edge. The flow topology was examined using schlieren photography and PIV. Long exposure schlieren images identified following flow structures such as jet induced bow shock, separation shock, Mach disc and leading edge shock. On the hand high speed schlieren visualisation revealed information concerning the large-scale structures that develop as the jet and crossflow stream interacted. Large eddies were found to reside in the shear layer at the periphery of the jet dominating nearfield flow structure. In terms of the separation region *CO*_2_ jet produced a bigger region and Helium jet has the smallest region.

PIV experiments provided detailed penetration, mixing and trajectory information of transverse jets. In the experiments only the transverse jet was seeded with powder particles and regulated, measurements were done at the centreline. Injected air, *CO*_2_ and Helium transverse jets were have quite different penetration trajectories even though they have the same momentum flux ratio, a parameter governing jet penetration. Helium has a very distinct behaviour at near field with minimal spreading. *CO*_2_ jet provided smaller penetration and Helium jet provided greater penetration compared to air jet. Analyses of ensembles of vector fields produced average features such as the penetration band and the information concerning fluctuations. Air jet exhibited medium penetration path and expanded substantially in farfield, whereas *CO*_2_ jet convected closer to the wall after a turn to main flow direction with the biggest spreading area. Helium jet bent abruptly after considerable vertical penetration and convects far from the wall. Turbulence intensity contours helped to identify the barrel shocks and Mach disc forming the initial jet boundary for the first time in open literature. Mach disc was clearly apparent with low turbulence region around the jet vicinity and its shape was similar for air and *CO*_2_ jets. For Helium jet the Mach disc could not be observed like in schlieren images and the turbulence level was higher at the jet core due to higher jet velocity. However in the farfield, the turbulence intensity level was found to be smaller compared to other gases in the core. Maximum turbulence occurred above the Mach disc due to the presence of the shear layer and at the intersection of the windward side of the barrel shock and bow shock.

## Figures and Tables

**Table 1.** Particle characteristics for air, *CO*_2_ and Helium transverse jets.

**Jet Gas**	***U****_jet_* **(m/s)**	***Kn****_d_*	***τ****_p_* ***μsec***	***τ****_f_* ***μsec***	**Sk**
*Air*	315	0.21	2.0	70	0.03
*CO*_2_	250	0.14	2.1	88	0.02
*Helium*	875	0.68	3.5	25	0.14

**Table 2.** Experimental test conditions.

**Jet Gas**	***p*_0_** **(*mbar*)**	***T*_0_** **(^0^*K*)**	***Re/m*** **·10^6^(1/*m*)**	***Re**_Djet_* **·10^3^**	***J***
*Air*	6490	375	13.0	46.5	2.7
*CO*_2_	6468	374	13.0	75.8	2.7
*Helium*	6520	373	13.2	16.0	2.7

	±0.7%	±2.0%	±3.5%	±2.8%	±4%

**Table 3.** Experimental and theoretical Mach disc heights (*h*) and penetration band (*H*) at *x/d_jet_* = 20.

**Jet Gas**	***h****_sch_* **(mm)**	***h****_PIV_* **(mm)**	***h****_theo_* **(mm)**	***H*** **at** ***x/d****_jet_* **= 20 (mm)**
*Air*	6.2 ± 0.2	6.4 ± 0.5	6.3	22.0 ± 1
*CO*_2_	6.2 ± 0.2	6.4 ± 0.5	6.5	26.4 ± 1
*He*	-	-	-	24.2 ± 1

**Table 4.** Uncertainty estimates.

**Test No**	***E*_<_***_U_***_>_*/U****_jet_*	$E_{\sqrt{<U^{\prime 2}>}}/U_{\text{jet}}$	***E****_cc_***/*U****_jet_*
*Air*	1.6%	1.3%	2.1%
*CO*_2_	1.5%	1.2%	2.7%
*He*	1.2%	0.9%	1.5%
